# Extracorporeal Shock Wave Therapy for Coronary Artery Disease: Relationship of Symptom Amelioration and Ischemia Improvement

**DOI:** 10.22038/aojnmb.2017.9899

**Published:** 2018

**Authors:** Youko Takakuwa, Masayoshi Sarai, Hideki Kawai, Akira Yamada, Kenji Shiino, Kayoko Takada, Yasuomi Nagahara, Meiko Miyagi, Sadako Motoyama, Hiroshi Toyama, Yukio Ozaki

**Affiliations:** 1 Department of Cardiology, School of Medicine, Fujita Health University, Toyoake, Aichi, Japan; 2 Department of Radiology, School of Medicine, Fujita Health University, Toyoake, Aichi, Japan

**Keywords:** Coronary artery disease, Myocardial perfusion image, Shock wave therapy

## Abstract

**Objective(s):**

The current management of coronary artery disease (CAD) relies on three major therapeutic options, namely medication, percutaneous coronary intervention (PCI), and coronary artery bypass grafting (CABG). However, severe CAD that is not indicated for PCI or CABG still bears a poor prognosis due to the lack of effective treatments. In 2006, extracorporeal cardiac shock wave (SW) therapy reported on human for the first time. This treatment resulted in better myocardial perfusion as evaluated by dipyridamole stress thallium scintigraphy, angina symptoms, and exercise tolerance. The aim of the present study was to investigate: myocardial perfusion images and evaluate the relationship between the ischemia improvement and symptom amelioration by SW therapy.

**Methods:**

We treated ten patients (i.e., nine males and one female) with cardiac SW therapy who had CAD but not indicated for PCI or CABG and aged 63–89 years old. After the SW therapy, all patients were followed up for three months to evaluate any amelioration of the myocardial ischemia based on symptoms, adenosine stress thallium scintigraphy, transthoracic echocardiography, and blood biochemical examinations.

**Results:**

The changes in various parameters were evaluated before and after cardiac SW therapy. The cardiac SW therapy resulted in a significant improvement in the symptoms as evaluated by the Canadian Cardiovascular Society [CCS] class score (P=0.016) and a tendency to improve in summed stress score (SSS) (P=0.068). However, no significant improvement was observed in the summed rest score (SRS), summed difference score (SDS), left ventricular wall motion score index (LVWMSI), N-terminal pro-brain natriuretic, and troponin I. The difference of CCS class score (ΔCCS) was significantly correlated with those of SSS (ΔSSS) and SDS (ΔSDS) (r=0.69, P=0.028 and r=0.70, P=0.025, respectively). There was no significant correlation between ΔCCS and other parameters. Furthermore, no significant difference was observed between the CCS improved and non-improved groups in terms of the baseline characteristics.

**Conclusion:**

The current study demonstrated the potential efficacy and safety of Cardiac SW therapy in CAD patients. As the findings indicated, symptom amelioration was associated with ischemia improvement by extracorporeal shock wave therapy for the CAD patients.

## Introduction

Coronary artery disease (CAD) is the leading cause of death worldwide, especially in developing countries (1). The current management of CAD relies on the improvement of lifestyle factors as well as three major therapeutic options, namely medication, percutaneous coronary intervention (PCI), and coronary artery bypass grafting (CABG). However, severe CAD patients who do not have an indication for PCI or CABG still have a poor prognosis due to the lack of effective treatments (2, 3).

Shock wave (SW) therapy has been widely used for lithotripsy or the treatment of certain orthopedic conditions, including bone fracture or calcific tendonitis (4, 5). In 2000, it was demonstrated that a low level of SW could upregulate vascular endothelial growth factor (VEGF), a strong mitogen that induces angiogenesis in human umbilical vascular endothelial cells in vitro (2, 3).

Accordingly, Shimokawa et al. demonstrated that a low level of SW enhanced the expression of VEGF and its receptor, namely Flt-1, in cultured human endothelial cells in vitro. In the mentioned study, the most effective level of SW was reported as 0.09 mJ/mm^2^, which is about 10% of that used for lithotripsy. In 2004, they reported that extracorporeal cardiac SW therapy induced neovascularization and improved myocardial ischemia in the in vivo porcine models of chronic myocardial ischemia without any adverse effects (6).

Based on such promising results from animal studies, in 2006, they performed cardiac SW therapy on nine patients with CAD, who had no indication for PCI or CABG. They performed cardiac SW therapy with 200 shots/spot at 0.09 mJ/mm^2^ for 20-40 spots three times a week/series. They observed a significant improvement in the symptoms, exercise tolerance, and myocardial perfusion as evaluated by dipyridamole stress thallium scintigraphy only in the ischemic myocardium where SW was applied.

The mentioned beneficial effects of SW therapy was reported to persist for at least 12 months, and no procedural complications or adverse effects were noted. Therefore, extracorporeal cardiac SW therapy could be regarded as a safe, effective, and non-invasive therapeutic strategy for severe CAD (2, 3).

In a study conducted in 2010, the patients were subjected to one series of placebo and SW therapy in a double-blind crossover manner with an interval of three months. The SW therapy was performed as 200 shots/spot at 0.09 mJ/mm^2^ for 40-60 spots per session entailed three sessions per week. On the other hand, the patients in the placebo group underwent the procedure of SW therapy without irradiation. After the completion of the therapy, the patients were followed up for three months.

In the mentioned clinical trial, low-energy SW therapy was reported to improve the symptoms (based on Canadian Cardiovascular Society [CCS] class score), exercise tolerance (based on the 6-min walking distance) and frequency of nitroglycerin usage. Furthermore, the left ventricular ejection fraction (LVEF) and LV stroke volume, evaluated by magnetic resonance imaging (MRI), were significantly improved with SW therapy (7).

Despite the efficacy of SW therapy as documented above, in some clinical cases, SW treatment improved ischemia without any improvement in symptoms, or it ameliorated the symptoms but not ischemia. In this regard, the important relationship between improvement of symptoms and that of ischemia has remained unclear yet. With this background in mind, the present study was conducted to investigate the myocardial perfusion images in detail and clarify the relationship between ischemia improvement and symptom amelioration.

## Methods

### Study population

This study was approved by the Ethical Committees of Fujita Health University Hospital (No: 12-210) on December 6, 2012. Informed consent was obtained from all patients. The inclusion and exclusion criteria are summarized in Table 1. In the three-year research period, 10 patients were investigated in this study, each of whom was followed up for three months.

**Table 1:** Inclusion and exclusion criteria of the study

Inclusion criteria	
1	Male and female over 20 years old
2	Even under adequate drug treatment in line with the guidelines, there are chest pain attacks
3	A case where there is no indication for existing treatment (catheter intervention or coronary artery bypass surgery), or a sufficient improvement effect can not be expected compared with risk
4	Patients of Class II to IV in the Canadian Heart Association (CCS) classification
5	A case in which there is a region where transient or constant ischemia occurs in diagnostic imaging such as stress myocardial scintigram or MRI

**Exclusion criteria**	

1	Patients who can not identify the target range with echocardiography or who can not focus shockwaves on the therapeutic target range
2	Patient who is transplanted with breast augmentation by silicone etc, the affected area is the region through which shock waves pass
3	Artificial valve (mechanical valve) patient after replacement surgery
4	Patient who is experiencing Q wave myocardial infarction within 3 months before shock wave treatment
5	Patients with non-Q wave myocardial infarction within 6 weeks before shock wave treatment
6	One month has not passed since the last coronary angioplasty/coronary artery bypass surgery
7	Patients with cardiogenic shock or cardiac insufficiency (patients requiring continuous infusion of cardiovascular drugs such as cardiotonic drugs and vasodilators)
8	A patient who has apparent cardiac thrombosis by echocardiography or ventricular angiography
9	Patient who changed angina pattern and clinical condition after the last coronary angiography examination
10	Diabetic retinopathy in which no control is available (cases with active fundus bleeding)
11	When malignant tumor coexists, or when you are undergoing surgery for malignant tumor within the past 5 years

We enrolled nine patients with severe angina pectoris who already underwent CABG or PCI and had further indications for therapy as well as one patient who underwent CAG, but had no indication for PCI due to the distal lesion. All 10 patients still suffered from stable effort angina under intensive medication. Diabetes was defined as fasting blood sugar of ≥ 126 mg/dL, blood sugar of ≥ 200 mg/dL, and HbA1c of > 6.5% during a 75-gram oral glucose tolerance test, or the use of antidiabetic drug(s).

Hypertension was determined as systolic blood pressure of > 140 mmHg, diastolic blood pressure of > 90 mmHg, or the use of antihypertensive drug(s). Furthermore, hypercholesterolemia was defined as low-density lipoprotein cholesterol of > 140 mg/dL or the use of lipid-lowering drug(s). All patients continued their oral medications during this study (2).

### Treatment protocol

**Figure 1:**
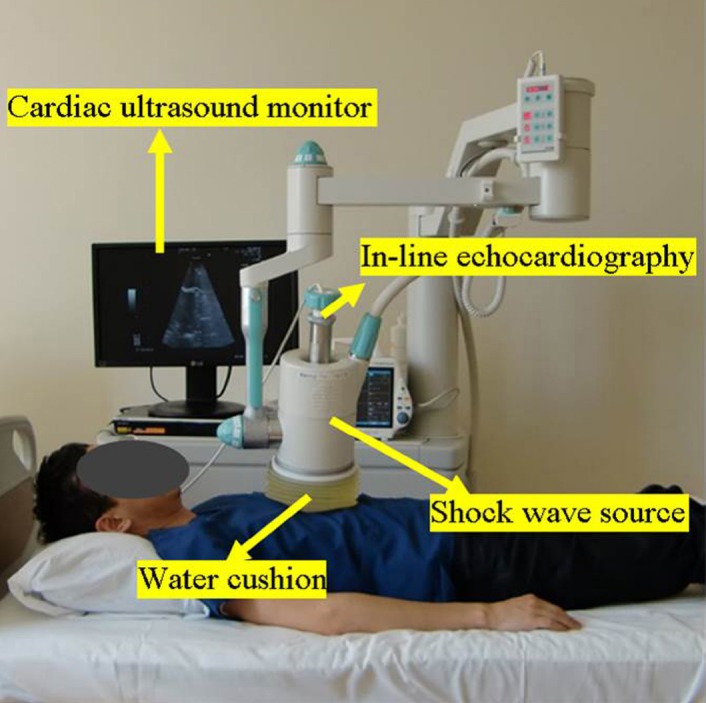
Implementation of cardiac shock wave therapy on a patient

We treated 10 patients with cardiac SW therapy (three times a week, 200 shoots/spot at 0.09 mJ/mm^2^ for 45 spots in the ischemic area each time, Modulith SLC, with electromagnetic SW source; Storz Medical, Kreuzlingen, Switzerland). As shown in Figure 1, the patient lay down on the bed in a supine position. No anesthesia was used during the therapy. Additionally, electrocardiography and the monitoring of blood pressure, respiration, and blood oxygen saturation were performed during the SW therapy each time.

(The machine was equipped with a shock wave generator and in-line echocardiography. The shock wave generator was attached to the chest wall of the patient. The shock wave pulse was easily focused on the ischemic myocardium under the guidance of echocardiography. There was no need of anesthesia or sedatives.)

The target myocardial regions and ischemic area were located by the echocardiography and single-photon emission computed tomography (SPECT), respectively. The SW was applied in an R-wave triggered manner to avoid inducing ventricular arrhythmias. In addition, 12-lead electrocardiography, blood chemistry testing (including troponin I and N-terminal pro-brain natriuretic peptide [NT-pro BNP]), and hematological analysis were performed before and after each session.

We followed up all patients for three months after the SW therapy to evaluate any change in myocardial ischemia based on symptoms, adenosine stress thallium scintigraphy, transthoracic echocardiography, and blood biochemistry examinations. The patients continued their oral medications throughout the study period. We evaluated the time course of symptoms with the CCS class scores (i.e., 1: ordinary physical activity, 2: slight limitation of ordinary activity, 3: marked limitation of ordinary activity, 4: inability to perform any activity without angina or angina at rest) (2).

### Adenosine stress thallium scintigraphy

All patients underwent adenosine stress myocardial SPECT/computed tomography (CT) imaging with thallium-201 (Tl-201) almost one month (mean: 18.5 days, range: 13.0-26.0 days) before and about three months (mean: 89.5 days, range: 87.5-91.5 days) after therapy. Pharmacological stress was induced by adenosine administered intravenously for 6 min (0.12 mg/kg/min). Furthermore, Tl-201 (111-148 MBq) was injected 3 min after the initiation of adenosine infusion.

Early and delayed images were acquired 15 min and 3 h after tracer injection, respectively. Myocardial SPECT/CT imaging was performed using a dual-detector gamma camera (Symbia T6/T16, Siemens AG, Munich, Germany). Delayed images were obtained with 17 views per head. CT for attenuation correction was performed after imaging. All SPECT images were obtained with the same method. The SPECT images were divided into 17 standardized myocardial segments defined by the American Heart Association.

Two observers determined semi-quantitative defect scores visually for each segment by mutual consent according to a scoring system (0: no defect, 1: mild defect, 2: moderate defect, 3: severe defect, 4: no uptake). Summed stress score (SSS) and summed rest score (SRS) were defined as the sum of the defect scores in early and delayed images, respectively. Summed difference score (SDS) was obtained by the subtraction of SRS from SSS.

### Rest echocardiography

The LV wall motion was evaluated by means of a 16-segment model (an 11.0-MHz Transducer; Philips iE33). A semi-quantitative wall motion scoring was performed by two independent blinded cardiologists with extensive experience in clinical echocardiography (i.e., more than five years). In this scoring system, the scores of 1, 2, 3, and 4 are given to normal or hyperkinetic, hypokinetic, akinetic, and dyskinetic or aneurysmal segments, respectively. The LV wall motion score index (LVWMSI) was calculated as the mean scores of all visualized segments (8, 9).

### Statistical analysis

Continuous variables were expressed as median (interquartile range). The differences between the continuous variables were analyzed using Wilcoxon’s signed-rank test. Furthermore, Spearman’s rank correlation coefficient was utilized for the evaluation of the relationship between the changes of continuous variables. Comparisons between the two groups were performed by using Wilcoxon rank-sum and Fisher’s exact tests. Statistical tests were two-tailed, and a p-value less than 0.05 was considered statistically significant. All statistical analyses were performed in the JMP software, version 12.2.0 (SAS Institute Inc., Cary, NC, USA).

## Results

### Patient selection

All the studied patients suffered from stable effort angina for more than one year and aged over 60 years. Out of 10 subjects, 9 cases were male, and 6 patients had a history of smoking. The participants had ischemic myocardium as documented by adenosine stress thallium scintigraphy, with no indication for PCI or CABG. Six patients previously underwent both PCI and CABG, two cases were subjected to PCI alone, one participant received CABG alone, and one subject underwent neither of the procedure. In addition, eight patients had old myocardial infarction, and all of them had at least two or more complications and were taking multiple medicines (Table 2). The patients’ cardiac events and hospital admission were not recorded during the observation period.

**Table 2:** Demographic characteristics and medical data of the patients

Case	Age (years)	Gender	Smoking history	Previous treatment	OMI	ASO	DM	HT	DLP	HD	Medications
1	64	M	+	None	-	-	-	+	+	-	AP, BB, CCB, S, N
2	72	M	-	CABG	+	+	+	+	-	-	AP, ARB, BB, N
3	73	M	+	CABG, PCI	+	-	-	+	+	-	AP, BB, CCB, S
4	63	M	+	CABG, PCI	-	+	+	+	+	+	AP, ARB, CCB, S, N
5	77	M	+	PCI	+	-	-	+	+	-	AP, ARB, CCB, BB, S, N
6	64	M	+	PCI	+	-	-	-	+	+	AP, BB, S, N
7	77	M	+	CABG, PCI	+	+	+	+	+	+	AP, ARB, CCB, BB, S, N
8	89	F	-	CABG, PCI	+	-	+	+	+	-	AP, ARB, BB, S, N
9	76	M	-	CABG, PCI	+	-	-	+	+	-	AP, BB, S, N
10	71	M	-	CABG, PCI	+	-	+	+	+	+	AP, ARB, BB, S, N

OMI: old myocardial infarction, ASO: arteriosclerosis obliterans, M: male, F: female, CABG; coronary artery bypass graft, PCI: percutaneous coronary intervention, DM: diabetes mellitus, HT: hypertension, DLP: dyslipidemia, HD: hemodialysis, AP: aspirin, BB: beta blocker, CCB: calcium channel blocker, S: statin, N: nitrate, ARB: angiotensin receptor blocker

### Effects of cardiac shock wave therapy

We examined the changes in various parameters before and after cardiac SW therapy. The cardiac SW therapy significantly improved the symptoms as evaluated by the CCS class score (P=0.016). SSS also tended to improve (P=0.068). Nonetheless, the SRS, SDS, LVWMSI, NT-proBNP, and troponin I did not show any meaningful improvement (Table 3).

**Table 3:** Parameters before and after cardiac shock wave therapy

Parameters	Before SW therapy	After SW therapy	P-value
CCS class	2 (2-3)	1 (1-2)	0.016
SSS	12 (7.8-20.5)	10.5 (4.8-16.3)	0.068
SRS	3 (1.8-11.3)	2.5 (0.0-8.5)	0.121
SDS	8.5 (5.0-12.3)	6.0 (4.0-9.0)	0.148
LVWMSI	1.3 (1.1-1.8)	1.2 (1.0-1.5)	0.188
NT-proBNP (pg/mL)	546 (125-7761)	897 (129-9924)	0.322
Troponin I (ng/mL)	0.023 (0.009-0.069)	0.029 (0.006-0.057)	0.641

CCS: Canadian Cardiovascular Society, SSS: summed stress score, SRS: summed rest score, SDS: summed difference score, LVWMSI: left ventricular wall motion score index, NT-proBNP: N-terminal pro brain natriuretic peptide

### Correlation between ΔCCS and other parameters

The changes of CCS class score (ΔCCS) significantly correlated with the changes of summed stress score (ΔSSS) and summed difference score (ΔSDS) (r=0.6881, P=0.0278 and r=0.6991, P=0.0245, respectively). However, ΔCCS showed no significant correlation with any of the other parameters (Table 4).

**Table 4:** Correlation between difference of Canadian Cardiovascular Society class score and other parameters

	ΔSSS	ΔSRS	ΔSDS	ΔLVWMSI	ΔNT-proBNP	ΔTropnin I
**ΔCCS class**	r=0.6881	r=0.5141	r=0.6991	r=-0.4402	r=0.1665	r=0.3026
	P=0.0278	P=0.1284	P=0.0245	P=0.2030	P=0.6458	P=0.3954

### Baseline characteristics of CCS non-improved and improved groups

Cases with ΔCCS of 0 and > 1 were categorized into a CCS non-improved (n=3) and improved (n=7) groups, respectively. No significant difference was observed between the two groups in terms of the baseline characteristics (Table 5).

**Table 5:** Baseline characteristics of Canadian Cardiovascular Society class non-improved and improved groups

Characteristics	Non-improved (n=3)	Improved (n=7)	P-value
Age, years (IQR)	77 (63-89)	72 (64-76)	0.566
Male, n (%)	2 (66.7%)	7 (100%)	0.300
Smoking history, n (%)	2 (66.7%)	4 (57.1%)	1.000
Previous PCI, n (%)	3 (100%)	5 (71.4%)	1.000
Previous CABG, n (%)	3 (100%)	4 (57.1%)	0.475
OMI, n (%)	2 (66.7%)	6 (85.7%)	1.000
ASO, n (%)	2 (66.7%)	1 (14.3%)	0.183
DM, n (%)	3 (100%)	2 (28.6%)	0.167
HT, n (%)	3 (100%)	6 (85.7%)	1.000
DLP, n (%)	3 (100%)	6 (85.7%)	1.000
HD, n (%)	2 (66.7%)	2 (28.6%)	0.500
Antiplatelet, n (%)	3 (100%)	7 (100%)	1.000
Statin, n (%)	3 (100%)	6 (85.7%)	1.000
BB, n (%)	2 (66.7%)	7 (100%)	0.300
CCB, n (%)	2 (66.7%)	3 (42.9%)	1.000
Nitrate, n (%)	3 (100%)	6 (85.7%)	1.000
ARB, n (%)	3 (100%)	3 (42.9%)	0.200

OMI: old myocardial infarction, ASO: arteriosclerosis obliterans, CABG; coronary artery bypass graft, PCI: percutaneous coronary intervention, DM: diabetes mellitus, HT: hypertension, DLP: dyslipidemia, HD: hemodialysis, BB: beta blocker, CCB: calcium channel blocker, ARB: angiotensin receptor blocker

### Parameters of CSS non-improved and improved groups

The two groups showed a significant difference regarding CCS (post-treatment), ΔCCS, ΔSSS, and ΔSDS (P=0.019, P=0.009, P=0.021, and P=0.030, respectively). However, there were no significant difference between the groups considering LVWMSI, NT-proBNP, and troponin I (Table 6).

**Table 6:** Parameters of Canadian Cardiovascular Society class non-improved and improved groups

Parameters	Non-improved(n=3)	Improved (n=7)	P-value
CCS class (pre-treatment)	2 (2-3)	2 (2-3)	1.000
CCS class (post-treatment)	2 (2-3)	1 (1-1)	0.019
ΔCCS class	0 (0-0)	1 (1-1)	0.009
SSS (pre-treatment)	14 (10-19)	11 (7-25)	0.648
SSS (post-treatment)	15 (14-21)	5 (4-15)	0.136
ΔSSS	-2 (-4-1)	4 (4-8)	0.021
SRS (pre-treatment)	5 (1-11)	3 (2-12)	1.000
SRS (post-treatment)	6 (0-13)	2 (0-8)	0.557
ΔSRS	-1 (-2-1)	2 (1-4)	0.064
SDS (pre treatment)	8 (5-13)	9 (5-12)	0.908
SDS (post treatment)	8 (8-15)	5 (1-7)	0.063
ΔSDS	-2 (-3-0)	3 (1-6)	0.030
LVWMSI (pre-treatment)	1.308 (1.154-1.692)	1.308 (1.000-2.000)	1.000
LVWMSI (post-treatment)	1.182 (1.000-1.615)	1.231 (1.000-1.462)	1.000
ΔLVWMSI	0.126 (0.077-0.154)	0.000 (0.000-0.153)	0.298
NT-proBNP (pre-treatment)	3114 (1414-40975)	173 (113-741)	0.068
NT-proBNP (post-treatment)	5826 (3809-22217)	196 (77-1047)	0.111
ΔNT-proBNP	-2395 (-2712-18758)	-6 (-697-27)	0.820
TnI (pre-treatment)	0.025 (0.020-0.076)	0.016 (0.007-0.067)	0.424
TnI (post-treatment)	0.033 (0.025-0.088)	0.008 (0.006-0.046)	0.418
ΔTnI	-0.012 (-0.013-0.000)	0.001 (-0.008-0.010)	0.209

### A representative example (Case 5)

A 77-year-old man had a history of myocardial infarction in the inferior wall. He suffered from diabetes mellitus, hypertension, and dyslipidemia. He was taking aspirin, angiotensin receptor blocker, calcium channel blocker, beta blocker, statin and nitrate. He was also ex-smoker. He had stenotic lesions in diagonal branch and periphery of the right coronary artery and left circumflex. From one month ago, chest pain at the time of exercise was recognized, and new lateral wall area ischemia and inferior wall infarction were confirmed by adenosine stress thallium scintigraphy.

**Figure 2:**
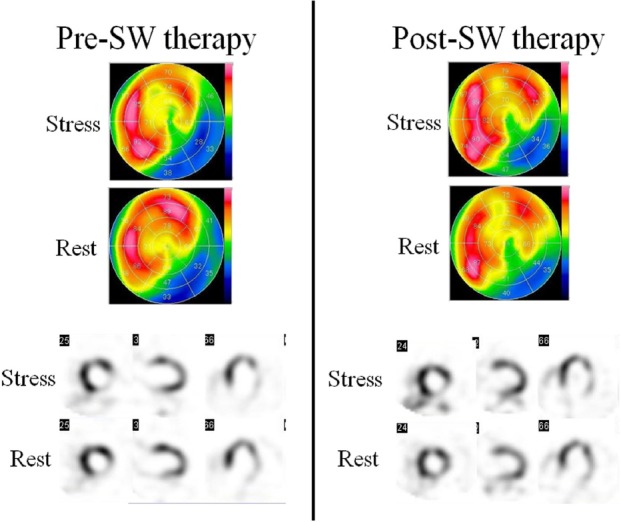
Stress myocardial perfusion scintigraphy of Case 5

His test results were CCS class II, SSS of 15, SRS of 11, SDS of 4, LVWMSI of 1.308, NT-proBNP of 113 pg/mL, and troponin I of less than 0.006 ng/mL. Due to the lack of indication for existing treatments, he underwent SW therapy for the lateral wall with ischemia. After SW therapy, both chest pain and lateral wall ischemia were improved. His test results after treatment were CCS class I, SSS of 10, SRS of 6, SDS of 4, LVWMSI of 1.182, NT-proBNP of 77 pg/mL, and troponin I of less than 0.006 ng/mL (Figure 2).

## Discussion

As the findings of the present study indicated, cardiac SW therapy could improve ischemic symptoms and reduce ischemic burden in patients with CAD who are already on optimal medical treatment. Moreover, the useful effects of this therapeutic approach were observed in the absence of detectable relevant side effects. We found that SW therapy was clinically feasible and successful in improving myocardial perfusion by SPECT and reducing the CCS score.

The findings of our study are consistent with the data presented in the literature, which demonstrated that cardiac SW therapy improves perfusion in ischemic settings and ameliorates ischemic symptoms (2, 7, 10). In line with the findings of a study (10), in the evaluation of myocardial ischemia by SPECT, although SSS showed an improving trend by cardiac SW therapy, there was no improvement in SRS or SDS. Regarding the cardiac function and cardiac markers, although LVWMSI, NT-proBNP, and troponin I were not improved by cardiac SW therapy in this study, some studies reported the improvement of the LV ejection fraction and systolic volume following SW therapy (7).

A noteworthy finding of this study was ΔCCS as an indicator of symptomatic improvement, which was significantly correlated with ΔSSS, and ΔSDS as indicators of ischemia amelioration. The possibility of the placebo effect accounting for the improvement in symptoms by cardiac SW therapy was also considered in this study. The findings of the present study revealed a relationship between the amelioration of symptoms and improvement of ischemia by cardiac SW therapy.

The comparison of the symptomatic improved group with non-improved group indicated no bias in the patient background. Regarding the parameters, although there was no difference in CCS before the treatment, a significant difference was observed in CCS and ΔCCS post-treatment. Among the ischemic indices, significant differences were found in ΔSSS and ΔSDS, revealing an improvement of blood flow abnormality during loading. According to the data obtained from animal experiments, this was due to extracorporeal cardiac SW therapy-induced neovascularization (11).

## Limitations of the Study

The present study has several limitations. The present study has no control arm for comparison. Furthermore, the sample size (n=10) was small, even though it took three years for us at a single institute to carefully enroll ten patients suitable for SW therapy and then follow them up for three months. To conduct a more thorough evaluation, the findings of the present study (i.e., the determination of the dose-dependent effect of the therapy) should be confirmed in a multicenter study using a much larger study population.

We were not able to perform a multivariate analysis due to our small sample size. However, we observed a correlation between the amelioration of anginal symptoms and improvement of ischemia. Moreover, we failed to evaluate exercise tolerance since 3 of the 10 patients had painful ambulation (i.e., one had an orthopedic condition, and the others were inflicted with arteriosclerosis obliterans). In addition, although the objectiveness is necessary for CCS decisions, the consideration of this issue was very difficult due to the retrospective nature of our research. However, the determination of CCS after SW therapy was performed prior to knowing the result of stress myocardial scintigraphy after treatment.

## Conclusion

The findings of the current study further demonstrated the potential efficacy and safety of cardiac SW therapy in CAD patients. After cardiac SW therapy, ischemic symptoms, and perfusion of CAD patients were significantly improved, and no SW complications were observed. As the findings revealed, symptom amelioration was associated with the improvement of ischemia in the CAD patients using extracorporeal SW therapy.
